# Stigmatisation of gambling disorder in social media: a tailored deep learning approach for YouTube comments

**DOI:** 10.1186/s12954-025-01169-0

**Published:** 2025-04-18

**Authors:** Johannes Singer

**Affiliations:** Gambling Research Center, University of Hohenheim, Schwerzstraße 44, 70599 Stuttgart, Germany

**Keywords:** Stigma, Self-stigma, Gambling, Gambling disorder, Personal responsibility, Social media, YouTube, Guided topic modelling

## Abstract

**Background:**

The stigmatisation of gamblers, particularly those with a gambling disorder, and self-stigmatisation are considered substantial barriers to seeking help and treatment. To develop effective strategies to reduce the stigma associated with gambling disorder, it is essential to understand the prevailing stereotypes. This study examines the stigma surrounding gambling disorder in Germany, with a particular focus on user comments on the video platform YouTube.

**Methods:**

The study employed a deep learning approach, combining guided topic modelling and qualitative summative content analysis, to analyse comments on YouTube videos. Initially, 84,024 comments were collected from 34 videos. After review, two videos featuring a person who had overcome gambling addiction were selected. These videos received significant user engagement in the comment section. An extended stigma dictionary was created based on existing literature and embeddings from the collected data.

**Results:**

The results of the study indicate that there is substantial amount of stigmatisation of gambling disorder in the selected comments. Gamblers suffering from gambling disorder are blamed for their distress and accused of irresponsibility. Gambling disorder is seen as a consequence of moral failure. In addition to stigmatising statements, the comments suggest the interpretation that many users are unaware that addiction develops over a period of time and may require professional treatment. In particular, adolescents and young adults, a group with a high prevalence of gambling-related disorders and active engagement with social media, represent a key target for destigmatisation efforts.

**Conclusions:**

It is essential to address the stigmatisation of gambling disorder, particularly among younger populations, in order to develop effective strategies to support treatment and help-seeking. The use of social media offers a comprehensive platform for the dissemination of information and the reduction of the stigmatisation of gambling disorder, for example by strengthening certain models of addiction.

## Background

A new State Treaty on Gambling entered into force in Germany in 2021. This first uniform federal regulation led to the liberalisation of the German gambling market by legalising previously illegal forms of gambling such as online sports betting and virtual slot machines [1]. The role of the state in protecting the population from harm caused by gambling is emphasised. The objective is to further reduce the prevalence of gambling disorders^1^ and ensure effective player protection, especially for children and young people. Recent surveys indicate that 2.4% of the German population suffers from a gambling-related disorder [5]. Negative consequences include financial problems, psychological distress, criminal activity [6] and even suicide [7]. The personal environment of affected persons, such as their partners and families, often suffers from the situation as well, as personal relationships are disrupted and the risk of domestic violence increases [7]. Gambling disorder is considered a behavioural addiction and is on a par with smoking, problematic drinking and recreational drug use [8]. Of particular concern is the fact that adolescents and young adults represent the group with the highest incidence of gambling disorder in Germany [5]. Among 18–25 year-olds, the prevalence reaches 4.9%, while among 26–35 year-olds, it stands at 3.7%. Therefore, protecting vulnerable groups from developing a gambling disorder is paramount and underlines the necessity of the German State Treaty’s objective to create conditions for effectively combating gambling addiction, for example through prevention strategies and therapy offers. Although gambling in Germany is only permitted from the age of 18, 0.4% of 16- to 17-year-olds suffer from a gambling disorder [5]. In order to mitigate the early onset of gambling-related harm among minors, it is essential to implement robust age verification processes and strictly enforce age restrictions, in addition to providing treatment and therapy.

The effects and consequences of gambling on individuals and society are a global phenomenon, with implications that extend across national boundaries. This conclusion was presented by the Lancet Public Health Commission on Gambling in a recent report [7], that clarified that gambling represents a global public health problem. Estimates suggest that in 2023, 46.2% of the adult population and 17.9% of adolescents worldwide were involved in gambling activities. The figures for possible gambling-related disorders varied between 0.4% and 1.7% for women and 1.8% and 4.1% for men, depending on the region [7].

In general, addictive disorders present significant challenges to public health, given that they are among the most stigmatised health conditions [9, 10]. The stigmatisation of addictive disorders can be categorised into two distinct forms: public stigma, which encompasses the negative attitudes and beliefs held by the general public towards a particular population group [11], and individual stigma, which reflects the perception of stigmatisation by the public on the part of the individual affected [12]. Both forms of stigma are problematic as they have been shown to be significant barriers to seeking treatment for people with addictive disorders [13–19]. In this regard, public perception of addictive disorders is of importance, as it influences the extent to which addictive disorders are associated with public stigma [20, 21]. Different models of addiction (MOAs) provide explanations for the development and maintenance of addictive disorders [20]. For instance, Rundle et al. [20] demonstrate, that the moral model results in increased stigmatisation of addictive disorders, as addiction is perceived as a moral transgression on the part of the individual affected. Conversely, the psychological MOA, which categorises addiction as psychological issue [22], can assist in reducing public stigma [20]. Thus, certain MOAs can contribute to the reduction of the stigma associated with addictive disorders.

Gambling disorder is often associated with stigma [23–26]. While existing studies clearly demonstrate the negative consequences of gambling disorder, research on stigmatisation is limited [25, 27]. In addition to the adverse effects on the individual’s personal well-being, people who suffer from a gambling disorder are also affected by social distancing [26, 28–31]. Moreover, the stigmatisation of gambling disorder is reflected in the fact that people affected by it are labelled with negative attributes. These stereotypes portray them as compulsive, impulsive, desperate, irresponsible, prone to risk-taking, depressed, greedy, irrational, anti-social, and aggressive [32]. In line with previous research indicating that addictive disorders are frequently perceived as moral failure [33–35], affected persons are blamed for their own situation. As noted by Miller and Thomas [36], the condemnation and stigmatisation of individuals with gambling disorder are based on the ascription of exclusive personal responsibility. Addictive behaviour is depicted as personal misconduct, attributed to inherent weakness, character flaws, and a lack of self-control [23, 24].

While most studies focus on the stigma associated with gambling disorder [36], Horch and Hodgins [37] showed that, in general, most forms of gambling are stigmatised to a certain extent, regardless of whether those affected are afflicted with a gambling disorder. In addition to its negative impact on personal life, public stigma can lead to self-stigmatisation [24, 38–40]. It is irrelevant whether those affected perceive public stigma, or whether they are directly or personally affected. In the process of self-stigmatisation, the affected person internalises prevailing negative attributions and prejudices [24, 39–42], referred to as individual stigma [21] or internalised stigma [12]. This results in the person adopting stereotypes, which causes additional psychological distress and can lead to lowered self-esteem. As a result, those affected blame themselves for their personal problems and tend to withdraw from their social environment. Those affected frequently encounter difficulties acknowledging their gambling disorder because of concerns about potential self-image deterioration [25]. This makes it challenging to seek available help and hinders the development of self-confidence in those affected, impending their ability to seek treatment [24]. Consequently, the stigmatisation of gambling disorder is considered a major barrier to treatment [25, 36, 39, 40, 43, 44], and also a cause of treatment discontinuation [45], as attending therapy can carry stigma itself [38].

To comply with the stipulations of the German State Treaty on Gambling [1], a fundamental objective of public health is to safeguard vulnerable populations from gambling disorder and diminish the prevalence of gambling-related disorder. Reducing stigma is a strategy to encourage more individuals to seek treatment [39, 40, 44, 46]. Increased awareness and a different perception of the MOA associated with gambling disorder among those affected by a gambling-related disorder, as well as the general public, can help reduce stigma [36, 39, 43]. Social media represents a promising channel for outreach. In Germany, where 60% of the population use social media at least once a week [47], this channel holds substantial potential. It is notable that among children, adolescents, and young adults aged 14 to 29 years, 92% engage with social networks on a weekly basis, with 62% accessing these networks on a daily basis [47]. However, these groups of young people are not only the most active users of social media [47], but also coincide with the groups with the highest prevalence of gambling disorder. The most affected age groups are those aged 18–25 and 26–35 [5].

A wide range of social networks, including Facebook, Instagram, TikTok, Twitter, and YouTube, have become a integral part of our daily lives. These platforms operate through user-generated content that may take the form of uploading pictures, sending tweets, or engaging in video commentary. The popularity of video platforms, particularly YouTube, has grown significantly in recent years. Currently, YouTube is the second largest social network in the world with over 2.5 billion monthly users [48]. In Germany, 81% of children and young people use this social network at least once a week [49]. It is important to note that social media has evolved beyond its role as a pure entertainment platform, becoming a dynamic space for information and interaction. Users actively seek to connect, exchange ideas and perspectives, and share their emotions.

Content from social media offers researchers nearly unlimited user-generated data that can be used in scientific research. Textual data can be extracted from social networks by leveraging appropriate Application Programming Interfaces (APIs). Due to the large amount of data, Natural Language Processing (NLP) has emerged as a useful tool for identifying the specific content of interest in text data. In addition to established topic modelling techniques, such as Latent Dirichlet Allocation (LDA), contemporary research is increasingly employing deep learning models. One such example is the Bidirectional Encoder Representations from Transformers, commonly referred to as BERT [50]. BERT is considered to be one of the most powerful NLP tools, because of its pre-trained word embedding model, which allows it to map more precise representations of words to sentences than comparable machine learning methods [51, 52]. For instance, BERT has been employed to analyse a range of content, including fake news [51, 53, 54], offensive language [55–58], online hate [59], sentiment [60, 61], racism, stigmatisation [62] and drug event detection [63] on social networks.

Previous studies have provided clear evidence of the nature and (negative) consequences of stigma associated with gambling disorder [26, 28–31]. Most studies are based on interviews or surveys to identify prevailing stereotypes [23, 24, 32, 36, 39, 64]. To our knowledge, however, there are no studies for Germany and no approaches that take social media into account. This study represents the first attempt to investigate stigmatisation associated with gambling disorder in social media by employing a deep learning approach. (i) After employing the deep learning approach, (ii) the results were validated using a qualitative summative content analysis and (iii) compared with the results from guided topic modelling. Finally, (iv) the categories of stigma associated with gambling disorder and supporting statements are discussed.

## Method

### Identification of YouTube videos

The first step is to select suitable videos whose content stimulates the exchange of users in the comments section and directs the conversation on the issue of gambling disorder. Therefore, videos are sought to (1) explicitly address gambling disorder and (2) feature a person who suffers from or has overcome a gambling disorder. For this purpose, five keywords, which were self-defined as in other research approaches [65, 66], were determined. The following keywords were identified as relevant to this study: *Gambling, sports betting, casino streams, gambling influencers, gambling addiction*. In addition, the following selection criteria were established: (3) the videos and comments must be in German, and (4) a video must have at least 1000 comments.

To avoid the YouTube algorithm from skewing the results based on the history of past searches it is necessary to conduct the search in a private browser window and perform the search without logging in to a YouTube account. The search was then performed separately for each keyword, and a list of the top 10 most viewed videos with at least  1000 comments was created. These lists were matched and duplicates were removed. This process yielded 34 distinct videos with 84,024 comments (see Appendix: Table 11). A subsequent review of the videos was conducted to ensure that a person who suffers from or has overcome a gambling disorder was the focus of the content. Two videos from the channel ‘Leroy wants to know!’^2^ were identified as being of particular interest. These videos are characterised by a high degree of seriousness, given that the channel is part of the content network of German public television called funk, represented by the first public channel (ARD^3^) and the second public channel (ZDF^4^). Both videos were comparatively up-to-date, with 11,813 comments in total. Although the videos are also available on the official website of ZDF^5^ and funk,^6^ only YouTube provides the functionality for users to post comments. In the first video, a person who has overcome a gambling disorder recounts his experiences of living with a gambling addiction. In the second video, a person who has overcome a gambling disorder engages in a discussion with a former casino owner about the moral implications and responsibilities associated with gambling. In both videos, the person who has overcome a gambling disorder is featured throughout the entire video.

### Data collection and processing

The YouTube Search Data APIv3 was leveraged to extract the title, URL, upload date, number of views, and comments of the selected videos, as well as the upload channel. This requires a Google account and a personalised access key to the YouTube API. Table 1 provides an overview of the data collected: 11,813 comments from two videos were collected on November 23, 2022.

**Table 1:** Information about the selected videos

Video title	Channel name^a^	URL	Upload date	Views (n)	Comments (n)	Gambling content^b^	Person with a gambling disorder^b^
GAMBLING ADDICT meets CASINO OWNER | The meeting	Leeroy will‘s wissen!	vYGEkC_0LX0	2022-04-07	3,454,674	6864	Yes (100%)	Yes (100%)
What is it like TO BE ADDICTED TO GAMBLING?	Leeroy will’s wissen!	PK_FTp4iHaQ	2020-08-31	1,470,656	4949	Yes (100%)	Yes (100%)

The data was collected at 23.11.2022. The video title was translated by the author. The videos are arranged in a chronological descending order based on their views

^a^Channel names have not been translated as they are proper names

^b^Indicate the percentage of the total duration of the video which is dedicated to gambling content, or alternatively, the length of time that the person is visible within the video

The deep learning approach, including pre-processing and guided topic modelling, was conducted using Python^7^ programming language and a range of packages.^8^ As part of the pre-processing, the text was converted to lower case, and German diacritics were converted to their non-diacritical combinations. URLs, punctuation, single letters, spaces, numbers and German stop words were excluded. Additionally, short words with fewer than three letters were removed to reduce noise in the data. A special tagger for the German language [71] was employed to lemmatise each word. Although the lemmatiser yields superior results to conventional stemmers, some words require manual correction. To minimise noise in the data, short sentences with fewer than ten words and rare words with fewer than 20 occurrences were also removed from the corpus after tokenisation. The final corpus for both videos consisted of 9451 tokens.

### Creation of an extended stigma dictionary

As a preliminary step in the process of guided topic modelling, a stigma dictionary was created. In the context of NLP, so-called dictionaries are indispensable components. They serve as the basis for the recognition of linguistic phenomena in textual data, including stigmas [72–74]. For this reason, a stigma dictionary is created in three steps that contains terms associated with the stigmatisation of gambling disorder. The initial step was to select words from the existing research. One limitation of previous studies is that they were based exclusively on interviews and surveys. It can be assumed that people express prevailing stereotypes differently in an anonymous setting, such as the comments section of YouTube, than in a research setting. A review of the data revealed that only three of the 19^9^ negative attributions identified in previous studies [32, 36, 39], were present in the data set under study: *foolish*, *naive*, and *stupid* (Table 2).

**Table 2:** Stigmatising terms from the literature found in the dataset

Terms	n
Foolish	40
Naïve	39
Stupid	225

For the purposes of the stigma dictionary, the terms were translated into German as follows: *blöd*, *naiv*, *dumm*

In the second step, four additional terms were added to the dictionary: *addiction*, *addict*, *gambling addicted* and *gambling addict*. As our approach is data-driven, we increased the basis on which embeddings can be used to search for terms that can be associated with the stigmatisation of gambling disorder. This is the third step. The use of embeddings is well established in NLP methods, such as the detection of hate speech on social media [75–77]. As elucidated by Young et al. [78], the supposition underlying the use of embeddings is that words with analogous meanings are situated in similar contexts. The utilisation of embeddings does not constrain the creation of a stigma dictionary to terms from the extant research literature; rather, it permits the identification of terms that may be associated with the stigmatisation of gambling disorder in the aggregated data. Table 3 illustrates the ten most frequent embeddings^10^ for the three existing negative attributions in our dataset and the four additional terms that have been added. Among these terms, those that exhibit similarities to the stereotypes identified in the literature are included in the stigma dictionary. For instance, the word *guilty* was selected because it can stigmatise gamblers with a gambling-related disorder by ascribing guilt to them. Other terms that cannot be associated with stigmatisation gambling disorder are disregarded. All terms in bold in Table 3 are included in the stigma dictionary.

**Table 3:** Embeddings of the stigmatising terms from the literature found in the dataset and the additional terms

Terms	Embeddings
Foolish	Intelligence, over, total, run, position, nothing, rich, broke, opinion, insight
Naïve	Nowadays, **guilty**, **weakness**, bad, **stupid**, just, **weak**, exist, rip off, possibly
Stupid	Smart, **guilt**, statement, complete, people, itch, person, no matter, such, **stupidity**
Addiction	**Gambling addicted**, bad, issue, year, environment, **gambling addicts**, video, drug addiction, **gambling addict**, problem
Addicted	**Guilt**, understand, casino, no matter, **responsibility**, casino owner, **responsible**, normal, people, owner
Gambling addicted	**Gambling addicts**, operator, **addiction**, casino, gambling hall, gamble away, casino owner, lose, **criminal**, **gambling addict**
Gambling addict	***Honest***, franklin, ***respect***, ***wish***, ***sympathetic***, speak, ***honesty***, interview, understand, ***strong***

The embeddings and terms were translated by the author

All terms in bold are included in the Stigma Dictionary

The stigma dictionary comprises three categories of terms: (1) terms that have been identified in existing research, (2) additional terms that explicitly refer to (gambling) addiction, and (3) terms from embeddings that can be associated with stigmatisation of gambling disorder. The aim was to supplement known stereotypes with negative attributions derived from the aggregated data. In this way, the utterances of users can be considered unfiltered, and it becomes clear how the stigmatisation of gamblers with a gambling-related disorder is produced in the everyday language of users. By extending the stigma dictionary, a total of 16 negative attributions were identified (Table 4). This extended stigma dictionary serves as a guideline for the guided topic modelling procedure carried out in the next step.

**Table 4:** Extended stigma dictionary

Terms from the literature (n = 3)	Additional terms (n = 4)	Embeddings (n = 9)
Foolish, naïve, stupid	Addiction, addicted, gambling addicted, gambling addict	Guilty, guilt, stupidity, responsible, responsibility, weak, weakness, criminal, gambling addicts

The embeddings of the term *gambling addict* indicates that not only are negative attributions expressed towards gambling disorder, but also supportive statements. The semantic field of the term contains positive attributes such as *honest*, *respect*, *wish*, *sympathetic*, *honesty* and *strong* (Table 3). This finding suggests that users want to support gamblers with a gambling-related disorder. A second dictionary was created to test this hypothesis (Table 5). The support dictionary comprising six terms, serves as an additional starting point for guided topic modelling.

**Table 5:** Support dictionary

Embeddings (n = 6)
Honest, respectful, desirable, sympathetic, honesty, strong

### Guided topic modelling with BERT

The application of machine learning techniques to the analysis of social media data is a valuable tool. NLP techniques, such as topic modelling approaches, facilitate the identification of topics within large amounts of text data. By incorporating additional information, in this case the extended stigma dictionary, the topic model is guided to search for specific content, that is all comments associated with the stigma of gambling disorder, and to place them in the same category. Currently, BERT^11^ represents one of the most powerful tools and demonstrates state-of-the-art performance in NLP tasks [50]. In contrast to other well-known methods, such as LDA (as in [80]), BERT is a deep neural network involving bi-directional transformers that implement attention mechanisms. This specific architecture enables accelerated training and concentration on the essence of texts. BERT pre-trains deep bi-directional representations of unlabelled text, thereby enabling more accurate words-to-sentences representations than comparable machine learning methods [51, 52]. BERT is a relatively new model, and to our knowledge, this is the first time that it has been applied to German text data from YouTube.

The initial stage of the proposed deep learning approach to analysing the stigmatisation of gambling disorder in YouTube comments involves guided topic modelling using BERT. By assigning negative (stigma) and positive (support) attributes to different seed topics, the model can assign specific keywords to different content categories [81]. BERT serves as a filter for the aggregated dataset by filtering all tokens that can be associated with the stigma of gambling disorder into a topic based on the extended stigma dictionary. A similar process was employed for the positive attributions and the support dictionary. In the second step, a qualitative summative content analysis was conducted to assess the plausibility of the assignment of each token to the stigma and support content categories.

Analysing social media data using machine learning methods is challenging. For NLP methods to function effectively, the data must undergo pre-processing. This is particularly evident in the sometimes slang-like German YouTube comments. Social media users do not adhere to standard language conventions, and language correction and lemmatisation are less effective in German than in English. Although short sentences of less than 10 words were removed during pre-processing, the remaining data were often still short sentences. As these have limited semantic content, BERT is not able to assign these tokens to a topic in a meaningful manner and marks them as outliers that cannot be assigned to any of the topics. For optimisation purposes, during the exploratory stages of our work, the following parameters yielded the best performance in reducing the number of outliers and avoiding the generation of identical topics (Table 6): *min_topic_size* was set to 30 to adjust the minimum size of a topic. This was done with the intention of minimising the number of outliers and including as many comments as possible in the model. A greater number of topics results in the creation of identical categories. Conversely, a smaller number of topics would preclude the possibility of defining categories defined by a limited number of tokens, potentially leading to their integration into other topics. Furthermore, the number of generated topics was constrained to 10 (*nr_topics* = 10) to divide the tokens into as few different categories as possible, while also preventing the generation of identical categories. Finally, the algorithm is permitted to form bigrams from individual tokens (*n_gram_range* = (1, 2)). This enables the examination of words used in combination, such as “gambling addiction”, rather than individual words alone. For example, bigrams enable our model to differentiate between gambling disorder in Topic 0 (“gambling addict”) and other addictions in Topic 4 (“addicted”; “alcohol”; “cigarette”) (Table 7).

**Table 6:** Optimal parameters for BERT

Parameter	min_topic_size	nr_topics	n_gram_range
Value	30	10	1,2

**Table 7:** Top terms per topic and match with the dictionaries

Topic	Keywords	Matching keywords with stigma dictionary (n = 16)	Matching keywords with support dictionary (n = 6)
0	**Guilt**, **guilty**, **responsibility**, **responsible**, human, money, **stupid**, **gambling addict**, people, moral	**6**	0
1	Euro, year, money, feel, addicted, win, gaming hall, cheesburger, life	1	0
2	Video, video video, interesting, super, format, interesting video, super video, gambling addict, channel, respect	1	1
3	Money, lose, human, invest, earn, addicted, gaming hall, gambler, win, gamble away	1	0
4	Alcohol, alcoholic, addicted, drink, guilt, human, casino, sell, responsible, cigarette	3	0
5	Youtubestreamer^a^, youtubeinfluencer^b^, youtube, youtubestreamer, youtuebstreamer, video, youtubeinfluencer youtubestreamer, youtubeinfluencer youtubeinfluencer, youtubereporter^c^, format, video youtubestreamer	0	0
6	Gambling addict, **sympathetic**, **hope**, gambling addict gambling addict, **wish**, **strong**, gambling addict sympathetic, gambling, make, sympathetic gambling addict	1	**4**
7	Channel, activate, bell, subscribe, channel activate, bell subscribe, activate bell, subscribe channel, topic, gladly channel	0	0
8	Algae^d^, algae algae, addiction, addicted, first, out, please, gamble away, hello, gaming hall	2	0
9	Format, interesting, super, cool format, cool^e^ format, cool^e^, cool, super format, format format, format interesting	0	0

The terms were translated by the author. Bigrams permit the naming of terms twice and the use of different combinations of words

^a^^−^^c^The terms reprsent pre-processed categories of proper names of corresponding streamers, influencers and reporters on YouTube

^d^The term is used to describe a symbol that is displayed on a slot machine game

^e^In German, the term ‘*geil’* is used. Its literal translation is ‘*horny’*, but in everyday language, it is used more like *‘cool’* or ‘*nice’*

### Qualitative summative content analysis

Due to known difficulties of machine learning methods in analysing data from social media, a qualitative summative content analysis [82] was performed to validate the findings of the guided topic modelling. For this purpose, all comments associated with the stigmatisation of gambling disorder (Topic 0) and the support for the person affected (Topic 6) were subjected to manual review. The qualitative analysis was carried out independently by two researchers, to ensure inter-rater reliability, and was based on the methodological guidelines of Hsieh and Shannon [82] for qualitative summative content analysis. The process is both inductive and deductive, as keywords can be defined before and during analysis. In this case, terms that have been previously identified as relevant to the stigmatisation of gambling disorder are taken up from the existing literature (stigma dictionary) and supplemented by corresponding embeddings from the aggregated dataset (extended stigma dictionary). All comments to be analysed were saved in an Excel spreadsheet for manual coding. Cohen’s κ [83] was calculated to measure the inter-rater reliability between the two researchers.

## Results

### Results of the deep learning approach

The results of the deep learning approach are listed in Table 7. For each of the ten topics generated, ten essential keywords can be seen. As specified in the hyperparameters (*nr_topics*), BERT filters the tokens of the aggregated dataset into ten topics. A look at the keywords suggests that Topic 0 can be associated with stigmatising gambling disorder, and Topic 6 with supporting gamblers with a gambling-related disorder (Table 7). The keywords in the topics matched the terms in the dictionaries created. Six of the ten terms in Topic 0 matched the stigma dictionary. Topic 6 contained four terms, whereas the support dictionary contained six terms. If we include the term *gambling addict*, there are even eight matches. In contrast, the other topics had comparatively fewer matches with the dictionaries (Table 7).

Following the completion of the Guided Topic Modelling process, BERT classified 5245 comments as outliers, indicating that they could not be assigned to a topic. The remaining 4206 tokens were filtered into the 10 created topics. As shown in Table 8, 850 comments can be assigned to the stigmatisation of gambling disorder. The number of tokens that can be attributed to the support of gamblers with a gambling-related disorder was 335. After excluding outliers, BERT sorts 28% of the comments in our aggregated dataset into two topics that we are seeking. Of these, BERT classified 20% of the comments as stigmatising and 8% as supportive.

**Table 8:** Stigmatisation and support for gambling disorder in the dataset

Topic	n	Proportion of classified comments (4206)
Stigmatisation	850	20%
Support	335	8%

### Results of the qualitative summative content analysis

A qualitative summative content analysis [82] was conducted to assess the viability of the guided topic modelling approach. A total of 1185 tokens from Topic 0 and Topic 6 were coded manually. This qualitative classification is based on knowledge about the stigma associated with addictive disorders, particularly gambling disorder. The results are of interest because they enable an analysis of the way in which users create such stereotypes in their everyday language use. However, they are primarily intended to serve the purpose of testing the categorisation of the guided topic modelling approach. To ensure inter-rater reliability, the comments were coded by a second researcher. Cohen’s κ was calculated to measure the inter-rater reliability [83]. Table 9 demonstrates a consistently high level of inter-rater reliability between the two researchers, with Cohen’s κ values of 0.92 and 0.98.

**Table 9:** Topics of the 1,185 classified comments

	BERTopic		Qualitative analysis			Agreement between BERTopic and the qualitative analysis
	n	%	n	%	Cohen’s κ^a^	%
Stigmatisation	850	20	666	16	0.92	78
Support	335	8	168	4	0.98	50

^a^The value for Cohen’s κ refer to the agreement of the two researchers regarding the qualitative coding of the 1185 comments identified by BERT as topics for stigmatisation and support

### Comparison of the guided topic modelling and the qualitative analysis

The results of the qualitative analysis indicated inaccuracies in the guided topic modelling process (Table 8). BERT filters 1185 tokens from the aggregated dataset into Topic 0 and Topic 6. The qualitative analysis revealed that only 666 tokens could be attributed to stigmatising gambling disorder, and only 168 tokens indicate support for gamblers with a gambling-related disorder. In comparison to the deep learning approach, the proportion of stigmatising comments in the total data set was thus 16% instead of 20%, while the proportion of supportive comments was 4% instead of 8%.

### Categories of stigma associated with gambling disorder and supporting statements

The results of the deep learning approach in combination with a qualitative summative content analysis offer valuable insights into the practices of users expressing themselves in the comment sections of the selected YouTube videos. The aggregated dataset reveals the presence of tokens that can be associated with both stigmatisation of gambling disorder and supportive expressions towards gamblers with a gambling-related disorder. For instance, users describe the person who has overcome a gambling disorder in the videos as sympathetic or strong. Furthermore, they also try to give him a sense of self-belief and hope that he will remain healthy.

Table 10 presents a series of illustrative examples of stigmatisation as identified by the BERT classification and qualitative summative content analysis. These examples demonstrate that stigma associated with gambling disorder manifests in various forms. On the one hand, there are personal insults, which label the person with gambling-related disorder as stupid, weak (Table 10, comment number 132), or not intelligent, and pejorative terms, such as loser or junkie. Other terms suggest that the person with gambling-related disorder lacks personal responsibility (Table 10, comment number 166). Some statements also point to the stereotype that individuals with gambling-related disorders attempt to shift blame to others for their situation instead of taking personal responsibility for their own lives (Table 10, comment number 1). Regarding personal responsibility, it is argued that it is not feasible to protect all individuals from potential harm to their health. Otherwise, the autonomy of other members of society would have to be constrained to an extensive degree (Table 10, comment number 684). Comparisons were made with other addictions such as alcohol, tobacco or shopping addiction. By making this comparison, some users marginalise the problem of gambling disorder and use this relativisation to place responsibility for developing a gambling disorder solely on the individual (Table 10, comment number 628). Some users even doubt that addiction exists (Table 10, comment number 294) and argue that the concept of addiction either serves as an excuse for those affected or deprives them of the opportunity to take responsibility for their own situation (Table 10, comment number 53). It is also important to note that a small proportion of users reported that they themselves suffer or have suffered from a gambling-related disorder. This reinforces the stereotypes described as a form of self-stigmatisation. Not only have the individuals internalised the prevailing stereotypes, but they also confront others with them (Table 10, comment number 568)).

**Table 10:** Examples of different categories of stigma associated with gambling disorder in the aggregated dataset

Comment number^a^	Content of the comment	Type of stigmatisation
132	*‘stupid and weak meets strong and smart, is not hate but that's just how I see it’*	Personal insult
166	*‘Mario*^*b*^* takes no responsibility for himself and his actions it seems’*	Not taking responsibility
1	*‘The gambler blames others instead of himself’*	Blaming others
53	*‘Those who play and become addicted have only themselves to blame’*	Own fault
294	*‘@Dr. D The fact that addiction is always and repeatedly seen as a disease is an absolute problem. With this argumentation, all responsibility is swept away. Addiction is a choice. You can't do anything for a disease. That is an immense difference. That's where I criticise all psychologists and doctors. It's terrible.’*	Gambling addiction is not an addictive disease
684	*‘Good that you say that. The thought came to me immediately. In the "self-determined" system that we want (with all its freedoms and duties), we have to reckon with outliers. Otherwise we would have to regulate everyone and that would take away our freedom.’*	Restriction of personal freedom
628	*‘I can understand both sides, but I would never blame an arcade owner, because at the end of the day there are so many addictions. Take shopping addiction, for example. Do you want to blame clothes manufacturers and ask them, "Why do you make clothes?’*	Relativising gambling addiction
568	*‘@Gloria Viktoria I was an addict myself and that's why I have this opinion. it's always your own decision if and how you do something. if you look for excuses afterwards and don't think that it was/is your fault, I think it just shows how weak the person is. and I don't even mean that in a bad way ^^ a simple example would be do you cheat on your partner because you feel the need to or don't do it because it's against your morals? a simple decision for everyone and yet some people do it. do you want to tell me that the person is not responsible for that?’*	Self-stigmatisation

The examples were corrected and translated by the author

^a^Comment number indicates the position of the comment in the whole dataset

^b^*Mario* is the name of the person in the two videos who suffered from a gambling disorder

## Discussion

This study contributes to existing research on stigmatisation of gambling disorder. User data provided by social media were used for the analysis of linguistic phenomena. By employing a deep learning approach, it is possible to identify statements that can be associated with the stigmatisation of gambling disorder in the aggregated data set derived from the comment section of two selected videos on the video platform YouTube. An extended stigma dictionary was created for analysis. In addition to terms from the existing literature, terms and their embeddings from the aggregated data set were used as the basis for identifying stigma associated with gambling disorder. Despite the differences between the guided topic model and qualitative summative content analysis, statements can be made about how stigmatising statements about gambling disorder are produced in the language used by users. For example, people are labelled with negative attributes, a lack of personal responsibility is attributed to them, or they are blamed for their situation (Table 10). In addition, some statements show a tendency towards self-stigmatisation. These findings are consistent with previous studies [24, 26, 32, 36] and show that the prevailing stereotypes are associated with moral judgements about the behaviour of gamblers with a gambling-related disorder.

The findings are cause for concern, as stigmatisation and self-stigmatisation are considered major barriers to the treatment of gambling-related disorder [25, 36, 37, 39, 40, 43, 44]. In Germany, the number of people with a gambling-related disorder is highest among adolescents and young adults [5]. The objective of public health care is to safeguard the population, particularly vulnerable groups, from the adverse effects of gambling. Social media represents a potential starting point in this regard, as children and young people in particular are the most active user group of social networks [84]. Social media has considerable potential as a tool for engaging with vulnerable groups. It offers a platform for raising awareness of the issue of stigma and gambling disorder, and thus contributes to the destigmatisation of gambling-related disorders.

Existing research has shown that perceptions of addictive disorders are linked to public perceptions of their development and maintenance [20]. Different MOAs can lead to varying levels of public stigma [20, 21, 85]. For example, the moral MOA leads to higher levels of stigma towards some addictive disorders, including gambling disorder [20]. Here, the cause of the disorder is attributed to voluntary choice and moral failing [17, 85–87], which is partly reflected in the comments from the aggregated data set that may be associated with the stigmatisation of gambling disorder (Table 10). However, there are also positive statements that do not accuse the person affected of moral failure and are meant to be supportive. As McGinty and Barry [88] showed, language can make an important contribution to reducing stigma, depending on how it is used to communicate with, define and educate others. Strengthening certain MOAs, such as the psychological MOA, can not only help reduce the public stigma of addictive disorders, including gambling disorder [20], but also prevent those affected from reinforcing existing stereotypes and experiencing self-stigmatisation [39, 89–91]. The destigmatisation and prevention of self-stigmatisation are important public health strategies to protect people, especially vulnerable groups, from public stigma and its negative consequences.

## Limitations and future directions

A limitation of the present study is the exclusive focus on a single social network, YouTube. The requirements of the video platform allow users to appear anonymously without their real-world names. Therefore, we cannot say who has watched the selected videos or who the authors of the individual comments are, as we do not have further information such as age or gender. This aspect may influence what users say, for example if they make a stigmatising comment or insult under the protection of anonymity. Future research should try to collect additional user data, such as demographic data, and consider other social networks to analyse the stigma associated with gambling disorder. In this way, more precise statements can be made and differences between genders and age groups can be identified. The same applies to language choice. While only German videos and comments were considered in this study, other languages can be included in the analysis of other studies.

Furthermore, the selection of videos was limited to one channel on YouTube, which is subsidised by the public broadcaster in Germany. Although we were not able to collect user data, it can be assumed that the audiences for different videos on different channels may differ, possibly also by language. It is also conceivable that attitudes may differ, for example in the perception of gambling addiction. Future approaches should take this into account.

The selection of YouTube videos was based on the consideration that individuals who have (overcome) a gambling disorder are featured for the entire duration. The videos were selected because their content stimulates user dialogue in the comments section and directs conversation towards the topic of gambling disorder. This results in a data frame with a sufficient number of 11,813 comments, which is reduced to 9451 tokens after pre-processing. Therefore, the two selected videos are not representative of other videos in which gamblers, for example, only appear as peripheral figures or other social networks. For future research, it would be beneficial to aggregate larger amounts of data in order to be able to make more representative statements about prevailing stereotypes associated with gambling disorder.

Overall, when analysing the stigmatisation of gambling disorder, it seems useful to consider different social networks, languages and larger amount of data. In particular, for the development of specific prevention strategies, it seems useful to examine different social networks to consider the specificities of the respective platforms. The different functions of social networks pose different challenges for the development of stigma reduction interventions, but at the same time offer different opportunities, such as raising awareness through different forms of communication including notifications, comments, pictures, or videos.

The language and some expressions used on social media present a challenge for the application of NLP techniques. This is because of the way users express themselves on social media, which often involves short sentences, sometimes single words or meaningless statements. By contrast, BERT is trained on long coherent sequences [52]. Speech recognition or lemmatisation packages often deliver comparatively poorer results in German than in English. Consequently, a significant proportion of terms and words had to be manually corrected during the pre-processing stage. Approximately half of the tokens could not be assigned to a specific topic and were classified as outliers; therefore, they were excluded from further analysis. This resulted in a reduction of the data set from 9451 comments after pre-processing to 4206 comments at the end. Further research could concentrate on the refinement of the pre-processing and optimisation of the model parameters, enhancing the informative value of the model and therefore reducing the number of outliers to a minimum extent. It would also be beneficial to analyse data from social media using so-called Large Language Models (LLMs), given that they deliver superior results when processing textual data in comparison to classic NLP models [92]. For example, these models are able to detect moral framings in textual data, as shown in a recent study by Sun and Fang [93]. If a more precise differentiation is possible, a distinction between different forms of stigma would be conceivable, for example between public stigma and self-stigmatising statements.

A qualitative summative content analysis was conducted to verify the plausibility of the results from the guided topic modelling process. This method is subject to a degree of subjectivity on the part of the researchers. To minimise this limitation, the coding of the data was carried out by two researchers and Cohen’s κ [83] was calculated, to measure inter-rater reliability. Although the comparison between the qualitative analysis and deep learning approach shows inaccuracies, with agreements of 78% for Topic 0 and 50% for Topic 6, the results are still interpretable. The primary aim of the study was not to quantify stigmatising statements, but rather to investigate how prejudices and stereotypes towards gambling disorder are created in the language used by users. However, only the comments were analysed using qualitative summative content analysis, which was classified in the first step using the deep learning model. In addition to improved NLP techniques, such as LLMs, to analyse larger amounts of text data, it would also be conceivable to use qualitative methods to obtain a detailed picture of stigmatisation on the one hand and supportive statements on the other. An in-depth understanding could be the basis for some destigmatisation approaches.

## Conclusions

This study represents a first approach for Germany to analyse the stigmatisation of gambling disorder in social media using a deep learning approach. The results of the guided topic modelling and qualitative summative content analysis demonstrate that deep learning methods, in this case BERT, are capable of identifying linguistic phenomena in text data. The model revealed the presence of various statements that can be associated with the stigmatisation of gambling disorder on the video platform YouTube. As demonstrated in previous studies [23–26, 36, 64], gambling disorder is associated with negative attributions and moral judgements. Future approaches could attempt to further optimise the model parameters to analyse as much semantic information as possible, by reducing the number of outliers. The use of Large Language Models could also be considered, as they have been shown to deliver superior results when processing text data compared to classic NLP methods [92].

Public understanding of how addictions develop and are maintained is crucial for the perception of addictive disorders [20]. Different MOAs may result in varying levels of stigmatisation [20, 21, 85]. The statements in the aggregated data set that can be associated with the stigma of gambling disorder exhibit similarities to the moral MOA, in that addiction is attributed to a moral failure on the part of the person affected [85]. Previous studies have demonstrated that the stigmatisation and self-stigmatisation associated with gambling disorder represents a significant barrier to treatment for those affected [25, 36, 37, 39, 40, 43, 44]. To address this issue, it is essential that public health plays a role in the implementation of effective prevention measures. Strengthening specific MOAs can lead to a change in the public perception of addictive disorders and, consequently, to a reduction in the stigma attached to gambling disorder. Reducing stigma also helps prevent self-stigmatisation of those affected by making it less likely that stereotypes will be internalised [39, 89–91]. As demonstrated by Rundle et al. [20], the psychological MOA can help reduce the stigma associated with addictive disorders, including gambling disorder. This is because it leads to a more compassionate and empathetic understanding of individuals struggling with a gambling disorder [21], as suggested by the supportive expressions in our aggregated data set. Furthermore, it is a public health responsibility to provide comprehensive treatment options and clearly communicate their availability and where to access them [94]. The dissemination of information via social media represents an appropriate channel for reaching the general public, particularly vulnerable groups. This further underscores the potential of this platform in contributing to the destigmatisation of gambling-related disorders. Those with a gambling-related disorder must be made aware that treatment is available, that recovery is possible [43] and that seeking help is not a sign of weakness but, above all, a sign of strength [24, 43].

## Data Availability

The datasets used and/or analysed during the current study are available from the corresponding author upon reasonable request.

## Appendix

See Table

**Table 11:** YouTube video information by keyword

Gambling							
Video title	Channel name^1^	URL	Upload date	Views (n)	Comments (n)	Gambling content	Person with a gambling disorder
Coin Master—Rip-off with FUN | NEO MAGAZIN ROYALE with Jan Böhmermann—ZDFneo	ZDF MAGAZIN ROYAL	hTeTjx4k9jQ	2019-10-10	3.469.481	4.923	Yes (100%)	No
KNOSSIS biggest BOOK OF DEAD WIN EVER!	Knossi	7vb5WpYNbvA^b^	2019-07-30	3.255.124	1.861	Yes (100%)	No
How rich will I get from gambling?—Self-experiment in a gambling hall	tomatolix	zvGFT1g35gc	2017-09-16	2.931.371	4.044 / 4.037	Yes (100%)	Yes (23%)
Gambling addiction—The business of gambling halls	Y-Kollektiv	GegsXxdH2zI	2017-01-12	1.844.735	1.689	Yes (100%)	Yes (22%)
Online gambling in Schleswig–Holstein | ZDF Magazin Royale	ZDF MAGAZIN ROYAL	9RV6i_zjoFI	2020-11-20	1.301.332	3.025	Yes (100%)	No
Gambling addiction: What makes gambling at the slot machine so dangerous? || PULS Reportage	PULS Reportage	KTTu1FZkIEs	2020-01-08	1.243.801	2.646	Yes (100%)	Yes (52%)
Elena LOSES at Gambling + Embarrassing Story | Talk with Elena	Cheasy	Rbuy_oX5-5g	2020-04-26	785.516	1.109	No	No
Online casino—How the gambling hype works on Twitch	Y-Kollektiv	3 × 8mIaaba0s	2019-12-05	616.705	2.152	Yes (100%)	Yes (10%)
1 week online casino—500€ turned into –€ | self-experiment	Tomary	Tj6TbacnbkQ	2020–05-09	654.732	1.240	Yes (100%)	Yes (12,5%)
40 h in the arcade: Do the staff intervene? | stern TV (2013)	stern TV	Cnok4vLIJJM	2022-08-23	509.553	1.091	Yes (100%)	No

Sports betting							
Video title	Channel name^1^	URL	Upload date	Views (n)	Comments (n)	Gambling content	Person with a gambling disorder
Exclusive: Members of the betting mafia spill the beans | STRG_F	STRG_F	Y79yUhdGhrU	2018-11-26	1.957.584	2.757 / 2.754	Yes (12%)	No
Money laundering at Tipico shops & co | STRG_F	STRG_F	GKJ3_bf9m8U	2019-06-11	1.093.583	1.710	No	No
Are sports bets dangerous? Meini vs. gambling || PULS Reportage	PULS Reportage	TyPSB-5CPJo	2020-10-28	458.358	1.135	Yes (100%)	Yes (30%)
I bought manipulated sports betting results on the Darknet!	Torben Platzer	wNEuSJIQYko	2022-10-30	439.446	1.346 / 1.347	No	No
Through SPORTS BETTING to the ROLEX | Trade Up ‘05 | @Dave	DAVE	AfNlH_rA4EY	2021–01-12	425.689	1.769	Yes (100%)	No
Getting rich through sports betting? (Matched Betting Experiment)	SELTIX	ZuKEcnN6_Eo	2021-02-27	417.000	1.575	Yes (100%)	No
Getting rich through betting experts? (experiment)	SELTIX	sD-Sl5BM9jg	2021-04-28	225.899	1.092	Yes (100%)	No

Casino streams							
Video title	Channel name^1^	URL	Upload date	Views (n)	Comments (n)	Gambling content	Person with a gambling disorder
ALGE ALGE! MEGA WINN! | RAZOR SHARK	Knossi	-NKbRwwWMIU^b^	2019-09-07	4.036.995	2.808	Yes (100%)	No
KNOSSIS biggest BOOK OF DEAD WIN EVER!	Knossi	7vb5WpYNbvA^b^	2019-07-30	3.255.124	1.861	Yes (100%)	No
MONTE donates €1000 to KNOSSI LIVE in STREAM!	Knossi	BUXW0bWxm_0^b^	2019-05-20	3.016.542	1.278	Yes (100%)	No
KNOSSI on a VISIT! Spider in the Gaming Room—Part 1 | MontanaBlack Stream Highlights	Die Crew	xRoQM60zXPE	2021-07-31	2.930.587	1.122	Yes (13%)	No
MONTANABLACK on times as a drug junkie, scandals, casino streams (Realtalk) & his drive (+ Yapi)	Tim Gabel	STD9LvUqGMI	2020-03-15	2.635.055	4.558 / 4557	Yes (12,5%)	No
MOST Twitch Subscribers Worldwide Live Cracked feat. Knossi | MontanaBlack Stream Highlights	Die Crew	3yD3olxiwI8	2019–09-02	2.534.139	3.046	No	No
Original XXL Rolex 12,000€ won at the casino MontanaBlack Stream Highlights	Die Crew	TtgBVvTZMhA	2019-06-19	1.188.397	1.965	Yes (37,5%)	No
RUNA RUNA! | Stream EXCALATES COMPLETELY | Book of the Dead	Knossi	1oFvhoIfji8^a^	2020-04-07	1.157.550	1.416	Yes (100%)	No
dangerous CASINO ADDICTION in GTA5 | Part 2 | SpontanaBlack	SpontanaBlack	_FfUg0UpZKA	2021-05-22	960.042	1.043	Yes (60%)	No
Casino streams regretted? Criticism of the community + call to KNOSSI MontanaBlack Realtalk	Richtiger Kevin	nc2pSJ61MMQ	2020-01-31	877.312	1.336	Yes (70%)	No

Gambling influencer							
Video title	Channel name^1^	URL	Upload date	Views (n)	Comments (n)	Gambling content	Person with a gambling disorder
Online casino—How the gambling hype works on Twitch	Y-Kollektiv	3 × 8mIaaba0s	2019-12-05	616.705	2.152	Yes (100%)	Yes (10%)
Online casinos: How Influencers Earn from the Addiction of Others | frontal	ZDFheute Nachrichten	3RoueSVblTI	2020-10-29	239.485	2.056	Yes (100%)	Yes (20%)

Gambling addiction							
Video title	Channel name^1^	URL	Upload date	Views (n)	Comments (n)	Gambling content	Person with a gambling disorder
GAMBLING ADDICT meets CASINO OWNER | The meeting	Leeroy will’s wissen!	vYGEkC_0LX0	2022-04-07	3.454.674	6.864	Yes (100%)	Yes (100%)
How rich will I get from gambling?—Self-experiment in a gambling hall	tomatolix	zvGFT1g35gc	2017-09-16	2.931.371	4.044	Yes (100%)	Yes (23%)
Gaming addiction in Korea | Galileo | ProSieben	Galileo	_C9KLfqIQx0	2016-07-16	2.676.917	7.418	No	No
In the GAMBLING HELL—The addiction to gambling—A look behind the scenes of the industry | HD Doku	WELT Doku	mFJGBL4uQiQ	2019-10-08	1.906.796	2.109 / 2.107	Yes (100%)	Yes (4%)
Gambling addiction—The business of gambling halls	Y-Kollektiv	GegsXxdH2zI	2017-01-12	1.844.735	1.689	Yes (100%)	Yes (22%)
What is it like TO BE ADDICTED TO GAMBLING?	Leeroy will’s wissen!	PK_FTp4iHaQ	2020-08-31	1.470.656	4.949	Yes (100%)	Yes (100%)
Gambling addiction: What makes gambling at the slot machine so dangerous? || PULS Reportage	PULS Reportage	KTTu1FZkIEs	2020-01-08	1.243.801	2.646	Yes (100%)	Yes (52%)
Addicted to Gambling with Hartz IV | Poor Germany | RTLZWEI Dokus	RTLZWEI Dokus	usvUxAa0rB0	2020-06-28	1.241.186	1.952	Yes (33%)	No
Gambling addiction—A self-experiment	maiLab	zARjpQF2WCs	2019-07-26	689.064	3.721	Yes (100%)	No
How 7 vs Wild helped me with my gambling addiction | Conclusion	RELOADIAK	SSZG8UA3hTQ	2020-01-08	597.450	1.231	Yes (10%)	Yes (10%)

The data was collected on 11/23/2022. The video title was translated by the author. The videos are arranged in a chronological descending order based on their number of views

^a^The channel names have not been translated as they are proper names

^b^The videos were no longer available at the time of last review on 07/23/2023

^11^.

## Footnotes

1 The existing literature uses the term *problem gambling*. Recent reports (such as [2] and [3]) recommend the use of other terms that do not themselves have a stigmatising effect. For this reason, this study uses the term *gambling disorder* rather than *problem gambling*, as in the (DSM-5 [4]).

2 The German name for the channel is ‚Leroy will’s wissen!‘

3 Working Group of the Public Broadcasting Authorities of the Federal Republic of Germany.

4 Second German Television.

5 Sample link to the second video in the ZDF media library: https://www.zdf.de/funk/leeroy-wills-wissen-12187/funk-update-spielsuechtiger-trifft-casinobesitzer---das-treffen-102.html

6 Sample link to the second video in the funk media library: https://play.funk.net/channel/leeroy-wills-wissen-12187/spielsuechtiger-trifft-casinobesitzer-das-treffen-1796297

7 The following version of Python was used during the work on this paper: 3.9.16.

8 Additional functions were used from the Gensim ([67]), NLTK ([68]), NumPy ([69]) and Pandas ([70]) packages.

9 The full list of negative attributions is as follows: *aggressive, anti-social, compulsive, depressed, desperate, stupid, greedy, impulsive, irrational, irresponsible, lazy, naïve, risk-taking, selfish, foolish, uncontrolled, unproductive, untrustworthy, weak-willed.*

10 The FastText ([79] package was used to find embeddings.

11 The following version of BERT was used during the work on this paper: 0.13.0.

## References

[1] Bundesländer. Staatsvertrag zur Neuregulierung des Glücksspielwesens in Deutschland: Glücksspielstaatsvertrag 2021—GlüStV 2021; 29.10.2020.

[2] Livingstone C, Rintoul A. Gambling-related suicidality: stigma, shame, and neglect. The Lancet Public Health. 2021;6:e4–5. 10.1016/S2468-2667(20)30257-7. 33417846 10.1016/S2468-2667(20)30257-7

[3] GambleAware. 12 ways to reduce stigma when discussing gambling harms—a language guide; 2023.

[4] Center for Behavioral Health Statistics and Quality. Impact of the DSM-IV TO DSM-5 changes on the national survey on drug use and health: Impact of the DSM-IV to DSM-5 Changes on the National Survey on Drug Use and Health. Rockville, Maryland; 2016.

[5] Buth S, Meyer G, Rosenkranz M, Kalke J. Glücksspielteilnahme und glücksspielbezogene Probleme in der Bevölkerung: Ergebnisse des Glücksspiel-Surveys 2023. Hamburg; 2023.

[6] Browne M, Langham E, Rawat V, Greer N, Li E, Rose J. Assessing gambling-related harm in Victoria: A public health perspective; 2016.

[7] Wardle H, Degenhardt L, Marionneau V, Reith G, Livingstone C, Sparrow M, et al. The lancet public health commission on gambling. The Lancet Public Health. 2024. 10.1016/S2468-2667(24)00167-1. 39491880 10.1016/S2468-2667(24)00167-1

[8] Browne M, Rawat V, Newall P, Begg S, Rockloff M, Hing N. A framework for indirect elicitation of the public health impact of gambling problems. BMC Public Health. 2020;20:1717. 10.1186/s12889-020-09813-z. 33198709 10.1186/s12889-020-09813-z PMC7670710

[9] Schomerus G, Lucht M, Holzinger A, Matschinger H, Carta MG, Angermeyer MC. The stigma of alcohol dependence compared with other mental disorders: a review of population studies. Alcohol Alcohol. 2011;46:105–12. 10.1093/alcalc/agq089. 21169612 10.1093/alcalc/agq089

[10] Room R, Rehm J, Trotter R II, Paglia A, Üstün TB. Cross-cultural views on stigma, valuation, parity and societal values towards disability. Disability and Culture: Universalism and Diversity; 2001. p. 247–91.

[11] Corrigan PW, Kuwabara SA, O’Shaughnessy J. The public stigma of mental illness and drug addiction. J Soc Work. 2009;9:139–47. 10.1177/1468017308101818.

[12] Smith LR, Earnshaw VA, Copenhaver MM, Cunningham CO. Substance use stigma: Reliability and validity of a theory-based scale for substance-using populations. Drug Alcohol Depend. 2016;162:34–43. 10.1016/j.drugalcdep.2016.02.019. 26972790 10.1016/j.drugalcdep.2016.02.019 PMC5293183

[13] Greiner MG, Dixon LB. The stigma of addiction. Psychiatr Serv. 2021;72:1105–6. 10.1176/appi.ps.72901. 34465203 10.1176/appi.ps.72901

[14] Volkow ND. Stigma and the toll of addiction. N Engl J Med. 2020;382:1289–90. 10.1056/NEJMp1917360. 32242351 10.1056/NEJMp1917360

[15] Tuliao AP, Holyoak D. Psychometric properties of the perceived stigma towards substance users scale: factor structure, internal consistency, and associations with help-seeking variables. Am J Drug Alcohol Abuse. 2020;46:158–66. 10.1080/00952990.2019.1658198. 31490713 10.1080/00952990.2019.1658198

[16] Gutierrez D, Crowe A, Mullen PR, Pignato L, Fan S. Stigma, help seeking, and substance use. TPC. 2020;10:220–34. 10.15241/dg.10.2.220.

[17] Fareed A. Evolution of opioid addiction as a brain disease from stigma to modern neurosciences. J Addict Dis. 2020;38:84–7. 10.1080/10550887.2019.1692619. 31762407 10.1080/10550887.2019.1692619

[18] Da Silveira PS, Casela ALM, Monteiro ÉP, Ferreira GCL, de Freitas JVT, Machado NM, et al. Psychosocial understanding of self-stigma among people who seek treatment for drug addiction. Stigma and Health. 2018;3:42–52. 10.1037/sah0000069.

[19] Luoma JB. Substance use stigma as a barrier to treatment and recovery. In: Johnson BA, editor. Addiction medicine. New York: Springer; 2011. p. 1195–215. 10.1007/978-1-4419-0338-9_59.

[20] Rundle SM, Goldstein AL, Wardell JD, Cunningham JA, Rehm J, Hendershot CS. Examining the relationship between public stigma, models of addiction, and addictive disorders. Addict Res Theory. 2024. 10.1080/16066359.2024.2365156. 10.1080/16066359.2024.2365156 PMC12124819 40453885

[21] Rundle SM, Goldstein AL, Wardell JD, Hendershot CS. Examining the relationship between acceptance of different models of addiction and individual stigma among outpatients with alcohol and opioid use disorders. Stigma and Health. 2024. 10.1037/sah0000550.

[22] Wiens TK, Walker LJ. The chronic disease concept of addiction: Helpful or harmful? Addict Res Theory. 2015;23:309–21. 10.3109/16066359.2014.987760.

[23] Horch JD, Hodgins DC. Public stigma of disordered gambling: social distance, dangerousness, and familiarity. J Soc Clin Psychol. 2008;27:505–28. 10.1521/jscp.2008.27.5.505.

[24] Carroll A, Rodgers B, Davidson T, Sims S. Stigma and help-seeking for gambling problems. 2013.

[25] Hing N, Holdsworth L, Tiyce M, Breen H. Stigma and problem gambling: current knowledge and future research directions. Int Gambl Stud. 2013;14:64–81. 10.1080/14459795.2013.841722.

[26] Hing N, Russell AMT, Nuske E, Gainsbury S. The stigma of problem gambling: causes, characteristics and consequences. 2015.

[27] Wöhr A, Wuketich M. Perception of gamblers: a systematic review. J Gambl Stud. 2021;37:795–816. 10.1007/s10899-020-09997-4. 33660191 10.1007/s10899-020-09997-4 PMC8364520

[28] Dhillon J, Horch JD, Hodgins DC. Cultural influences on stigmatization of problem gambling: East Asian and Caucasian Canadians. J Gambl Stud. 2011;27:633–47. 10.1007/s10899-010-9233-x. 21203804 10.1007/s10899-010-9233-x

[29] Palmer BA, Richardson EJ, Heesacker M, DePue MK. Public stigma and the label of gambling disorder: Does it make a difference? J Gambl Stud. 2018;34:1281–91. 10.1007/s10899-017-9735-x. 29243011 10.1007/s10899-017-9735-x

[30] Peter SC, Li Q, Pfund RA, Whelan JP, Meyers AW. Public stigma across addictive behaviors: casino gambling, esports gambling, and internet gaming. J Gambl Stud. 2019;35:247–59. 10.1007/s10899-018-9775-x. 29627881 10.1007/s10899-018-9775-x

[31] Hing N, Russell AMT, Gainsbury SM. Unpacking the public stigma of problem gambling: the process of stigma creation and predictors of social distancing. J Behav Addict. 2016;5:448–56. 10.1556/2006.5.2016.057. 27513611 10.1556/2006.5.2016.057 PMC5264412

[32] Horch J, Hodgins D. Stereotypes of problem gambling. JGI. 2013;66:1. 10.4309/jgi.2013.28.10.

[33] Rise J, Halkjelsvik T. Conceptualizations of addiction and moral responsibility. Front Psychol. 2019;10:1483. 10.3389/fpsyg.2019.01483. 31316438 10.3389/fpsyg.2019.01483 PMC6610207

[34] Frank LE, Nagel SK. Addiction and moralization: the role of the underlying model of addiction. Neuroethics. 2017;10:129–39. 10.1007/s12152-017-9307-x. 28725284 10.1007/s12152-017-9307-x PMC5486499

[35] Berridge KC. Is addiction a brain disease? Neuroethics. 2017;10:29–33. 10.1007/s12152-016-9286-3. 28706571 10.1007/s12152-016-9286-3 PMC5503469

[36] Miller HE, Thomas S. The, “Walk of Shame”: a qualitative study of the influences of negative stereotyping of problem gambling on gambling attitudes and behaviours. Int J Ment Heal Addict. 2017;15:1284–300. 10.1007/s11469-017-9749-8.

[37] Horch JD, Hodgins DC. Self-stigma coping and treatment-seeking in problem gambling. Int Gambl Stud. 2015;15:470–88. 10.1080/14459795.2015.1078392.

[38] Corrigan P. How stigma interferes with mental health care. Am Psychol. 2004;59:614–25. 10.1037/0003-066X.59.7.614. 15491256 10.1037/0003-066X.59.7.614

[39] Hing N, Nuske E, Gainsbury SM, Russell AM. Perceived stigma and self-stigma of problem gambling: perspectives of people with gambling problems. Int Gambl Stud. 2016;16:31–48. 10.1080/14459795.2015.1092566.

[40] Hing N, Russell AMT. How anticipated and experienced stigma can contribute to self-stigma: the case of problem gambling. Front Psychol. 2017;8:235. 10.3389/fpsyg.2017.00235. 28270787 10.3389/fpsyg.2017.00235 PMC5318456

[41] Corrigan PW, Watson AC. The paradox of self-stigma and mental illness. Clin Psychol Sci Pract. 2002;9:35–53. 10.1093/clipsy.9.1.35.

[42] Corrigan PW, Watson AC, Barr L. The self-stigma of mental illness: implications for self-esteem and self-efficacy. J Soc Clin Psychol. 2006;25:875–84. 10.1521/jscp.2006.25.8.875.

[43] Brown KL, Russell AMT. Exploration of intervention strategies to reduce public stigma associated with gambling disorder. J Gambl Stud. 2019. 10.1007/s10899-019-09888-3. 10.1007/s10899-019-09888-3 31440874

[44] Hing N, Nuske E, Gainsbury SM, Russell AM, Breen H. How does the stigma of problem gambling influence help-seeking, treatment and recovery? A view from the counselling sector. Int Gambl Stud. 2016;16:263–80. 10.1080/14459795.2016.1171888.

[45] Sirey JA, Bruce ML, Alexopoulos GS, Perlick DA, Raue P, Friedman SJ, Meyers BS. Perceived stigma as a predictor of treatment discontinuation in young and older outpatients with depression. Am J Psychiatry. 2001;158:479–81. 10.1176/appi.ajp.158.3.479. 11229992 10.1176/appi.ajp.158.3.479

[46] Hing N, Russell AMT. Psychological factors, sociodemographic characteristics, and coping mechanisms associated with the self-stigma of problem gambling. J Behav Addict. 2017;6:416–24. 10.1556/2006.6.2017.056. 28849669 10.1556/2006.6.2017.056 PMC5700730

[47] Müller T. Zahl der social-media-nutzenden steigt auf 60 Prozent. Media Perspektiven. 2024;2024:1–8.

[48] We Are Social, DataReportal, Meltwater. Ranking der größten Social Networks und Messenger nach der Anzahl der Nutzer im Februar 2025 (in Millionen). 2025. https://de.statista.com/statistik/daten/studie/181086/umfrage/die-weltweit-groessten-social-networks-nach-anzahl-der-user/. Accessed 13 Mar 2025.

[49] Kupferschmitt T. Aktuelle Ergebnisse der repräsentativen Studie: ARD/ZDF-Medienstudie 2024: Trends bei Video- und Audioplattformen. Media Perspektiven. 2024:1–9.

[50] Devlin J, Chang M-W, Lee K, Toutanova K. BERT: pre-training of deep bidirectional transformers for language understanding: arXiv; 2018.

[51] Kaliyar RK, Goswami A, Narang P. FakeBERT: Fake news detection in social media with a BERT-based deep learning approach. Multimed Tools Appl. 2021;80:11765–88. 10.1007/s11042-020-10183-2. 33432264 10.1007/s11042-020-10183-2 PMC7788551

[52] Devlin J, Chang M-W, Lee K, Toutanova K. BERT: pre-training of deep bidirectional transformers for language understanding. In: Burstein J, Doran C, Solorio T, editors. Proceedings of the 2019 Conference of the North; Minneapolis, Minnesota. Stroudsburg, PA, USA: Association for Computational Linguistics; 2019. pp. 4171–4186. 10.18653/v1/N19-1423.

[53] Singhal S, Shah RR, Chakraborty T, Kumaraguru P, Satoh S. SpotFake: a multi-modal framework for fake news detection. In: 2019 IEEE Fifth International Conference on Multimedia Big Data (BigMM); 11.09.2019 - 13.09.2019; Singapore, Singapore: IEEE; 2019. pp. 39–47. 10.1109/BigMM.2019.00-44.

[54] Tacchini E, Ballarin G, Della Vedova ML, Moret S, de Alfaro L. Some like it hoax: automated fake news detection in social. Networks. 2017. 10.48550/arXiv.1704.07506.

[55] Pitsilis GK, Ramampiaro H, Langseth H. Detecting offensive language in tweets using deep. Learning. 2018. 10.48550/arXiv.1801.04433.

[56] Aggarwal P, Horsmann T, Wojatzki M, Zesch T. LTL-UDE at SemEval-2019 Task 6: BERT and two-vote classification for categorizing offensiveness. In: May J, Shutova E, Herbelot A, Zhu X, Apidianaki M, Mohammad SM, editors. Proceedings of the 13th International Workshop on Semantic Evaluation; Minneapolis, Minnesota, USA. Stroudsburg, PA, USA: Association for Computational Linguistics; 2019. pp. 678–682. 10.18653/v1/S19-2121.

[57] Zampieri M, Malmasi S, Nakov P, Rosenthal S, Farra N, Kumar R. SemEval-2019 Task 6: identifying and categorizing offensive language in social media (OffensEval): arXiv; 2019.

[58] Liu P, Li W, Zou L. NULI at SemEval-2019 Task 6: Transfer learning for offensive language detection using bidirectional transformers. In: May J, Shutova E, Herbelot A, Zhu X, Apidianaki M, Mohammad SM, editors. Proceedings of the 13th International Workshop on Semantic Evaluation; Minneapolis, Minnesota, USA. Stroudsburg, PA, USA: Association for Computational Linguistics; 2019. pp. 87–91. 10.18653/v1/S19-2011.

[59] Salminen J, Hopf M, Chowdhury SA, Jung S, Almerekhi H, Jansen BJ. Developing an online hate classifier for multiple social media platforms. Hum Cent Comput Inf Sci. 2020. 10.1186/s13673-019-0205-6.

[60] Wang T, Lu K, Chow KP, Zhu Q. COVID-19 sensing: negative sentiment analysis on social media in China via BERT model. IEEE Access. 2020;8:138162–9. 10.1109/ACCESS.2020.3012595. 34812342 10.1109/ACCESS.2020.3012595 PMC8545339

[61] Yulita IN, Wijaya V, Rosadi R, Sarathan I, Djuyandi Y, Prabuwono AS. Analysis of government policy sentiment regarding vacation during the COVID-19 pandemic using the bidirectional encoder representation from transformers (BERT). Data. 2023;8:46. 10.3390/data8030046.

[62] Pei X, Mehta D. Multidimensional racism classification during COVID-19: stigmatization, offensiveness, blame, and exclusion. Soc Netw Anal Min. 2022;12:131. 10.1007/s13278-022-00967-9. 36090694 10.1007/s13278-022-00967-9 PMC9451118

[63] Fan B, Fan W, Smith C, Garner H. Adverse drug event detection and extraction from open data: a deep learning approach. Inf Process Manag. 2020;57:102131. 10.1016/j.ipm.2019.102131.

[64] Hing N, Russell A, Gainsbury S, Nuske E. The public stigma of problem gambling: its nature and relative intensity compared to other health conditions. J Gambl Stud. 2016;32:847–64. 10.1007/s10899-015-9580-8. 26487344 10.1007/s10899-015-9580-8 PMC4993796

[65] Obadimu A, Khaund T, Mead E, Marcoux T, Agarwal N. Developing a socio-computational approach to examine toxicity propagation and regulation in COVID-19 discourse on YouTube. Inf Process Manage. 2021;58:102660. 10.1016/j.ipm.2021.102660. 10.1016/j.ipm.2021.102660 PMC9759669 36567973

[66] Sánchez PRP, Folgado-Fernández JA, Sánchez MAR. Virtual reality technology: analysis based on text and opinion mining. Math Biosci Eng. 2022;19:7856–85. 10.3934/mbe.2022367. 35801447 10.3934/mbe.2022367

[67] Řehůřek R, Sojka P. Gensim–python framework for vector space modelling; 2011.

[68] Bird S, Klein E, Loper E. Natural language processing with Python. Sebastopol, California: O’Reilly; 2009.

[69] Harris CR, Millman KJ, van der Walt SJ, Gommers R, Virtanen P, Cournapeau D, et al. Array programming with NumPy. Nature. 2020;585:357–62. 10.1038/s41586-020-2649-2. 32939066 10.1038/s41586-020-2649-2 PMC7759461

[70] McKinney W. Data structures for statistical computing in python. In: Python in Science Conference; June 28–July 3 2010; Austin, Texas: SciPy; 2010. p. 56–61. 10.25080/Majora-92bf1922-00a.

[71] Wartena C. A probabilistic morphology model for German lemmatization. In: Proceedings of the 15th Conference on Natural Language Processing (KONVENS 2019); 2019. p. 40–49. 10.25968/opus-1527.

[72] Gottipati S, Chong M, Kiat A, Kawidiredjo B. Exploring media portrayals of people with mental disorders using NLP. In: 14th International Conference on Health Informatics; 11.02.2021–13.02.2021; Online Streaming—Select a Country—SCITEPRESS—Science and Technology Publications; 2021. p. 708–715. 10.5220/0010380007080715.

[73] Oscar N, Fox PA, Croucher R, Wernick R, Keune J, Hooker K. Machine learning, sentiment analysis, and tweets: an examination of Alzheimer’s disease stigma on twitter. J Gerontol B Psychol Sci Soc Sci. 2017;72:742–51. 10.1093/geronb/gbx014. 28329835 10.1093/geronb/gbx014

[74] Pokharel R, Bhatta D. Classifying YouTube comments based on sentiment and type of sentence: arXiv; 2021.

[75] Djuric N, Zhou J, Morris R, Grbovic M, Radosavljevic V, Bhamidipati N. Hate Speech Detection with Comment Embeddings. In: Gangemi A, Leonardi S, Panconesi A, editors. WWW ‘15: 24th International World Wide Web Conference; 18 05 2015 22 05 2015; Florence Italy. New York, NY, USA: ACM; 2015. p. 29–30. 10.1145/2740908.2742760.

[76] Nobata C, Tetreault J, Thomas A, Mehdad Y, Chang Y. Abusive Language Detection in Online User Content. In: Bourdeau J, Hendler JA, Nkambou RN, Horrocks I, Zhao BY, editors. WWW ‘16: 25th International World Wide Web Conference; 11 04 2016 15 04 2016; Montréal Québec Canada. Republic and Canton of Geneva, Switzerland: International World Wide Web Conferences Steering Committee; 2016. p. 145–153. 10.1145/2872427.2883062.

[77] Salminen J, Almerekhi H, Milenković M, Jung S, An J, Kwak H, Jansen B. Anatomy of online hate: developing a taxonomy and machine learning models for identifying and classifying hate in online news media. ICWSM. 2018. 10.1609/icwsm.v12i1.15028.

[78] Young T, Hazarika D, Poria S, Cambria E. Recent trends in deep learning based natural language processing [review article]. IEEE Comput Intell Mag. 2018;13:55–75. 10.1109/MCI.2018.2840738.

[79] Bojanowski P, Grave E, Joulin A, Mikolov T. Enriching Word Vectors with Subword Information: arXiv; 2016.

[80] Blei DM, Ng AY, Jordan MI. Latent dirichlet allocation. J Mach Learn Res. 2003;3:993–1022.

[81] Grootendorst M. BERTopic: Neural topic modeling with a class-based TF-IDF procedure: arXiv; 2022.

[82] Hsieh H-F, Shannon SE. Three approaches to qualitative content analysis. Qual Health Res. 2005;15:1277–88. 10.1177/1049732305276687. 16204405 10.1177/1049732305276687

[83] Cohen J. A coefficient of agreement for nominal scales. Educ Psychol Measur. 1960;20:37–46. 10.1177/001316446002000104.

[84] Koch W. Soziale medien werden 30 minuten am tag genutzt—Instagram ist die plattform Nummer eins. Media Perspekt. 2023;2023:1–8.

[85] Rundle SM, Cunningham JA, Hendershot CS. Implications of addiction diagnosis and addiction beliefs for public stigma: a cross-national experimental study. Drug Alcohol Rev. 2021;40:842–6. 10.1111/dar.13244. 33493359 10.1111/dar.13244

[86] Volkow ND, Koob G. Brain disease model of addiction: Why is it so controversial? Lancet Psychiatry. 2015;2:677–9. 10.1016/S2215-0366(15)00236-9. 26249284 10.1016/S2215-0366(15)00236-9 PMC4556943

[87] Volkow ND, Gordon JA, Koob GF. Choosing appropriate language to reduce the stigma around mental illness and substance use disorders. Neuropsychopharmacol. 2021;46:2230–2. 10.1038/s41386-021-01069-4. 10.1038/s41386-021-01069-4 PMC8580983 34276051

[88] McGinty EE, Barry CL. Stigma reduction to combat the addiction crisis—developing an evidence base. N Engl J Med. 2020;382:1291–2. 10.1056/NEJMp2000227. 32242352 10.1056/NEJMp2000227

[89] Nerilee Hing, Elaine Nuske, Sally Gainsbury. Gamblers at risk and their help-seeking behaviour: Unpublished; 2012.

[90] Delfabbro PH. Australasian gambling review. 5th ed. Rundle Mall, S. Aust.: Independent Gambling Authority; 2012.

[91] Cunningham JA. Little use of treatment among problem gamblers. Psychiatr Serv. 2005;56:1024–5. 10.1176/appi.ps.56.8.1024-a. 16088027 10.1176/appi.ps.56.8.1024-a

[92] Demszky D, Yang D, Yeager DS, Bryan CJ, Clapper M, Chandhok S, et al. Using large language models in psychology. Nat Rev Psychol. 2023. 10.1038/s44159-023-00241-5.

[93] Sun M, Fang Y. Unraveling the impact of moral framings within media coverage to promote the (de)stigmatization of depression on social media. J Health Commun. 2024;29:672–81. 10.1080/10810730.2024.2411320. 39376089 10.1080/10810730.2024.2411320

[94] Livingstone C, Rintoul A, Lacy-Vawdon C de, Borland R, Dietze PM, Jenkinson R, et al. Identifying effective policy interventions to prevent gambling-related harm; 2019.

